# Social Isolation in Older Adults: A Qualitative Study on the Social Dimensions of Group Outdoor Health Walks

**DOI:** 10.3390/ijerph19095353

**Published:** 2022-04-28

**Authors:** Katherine N. Irvine, Daniel Fisher, Melissa R. Marselle, Margaret Currie, Kathryn Colley, Sara L. Warber

**Affiliations:** 1 Social, Economic and Geographical Sciences Department, James Hutton Institute, Aberdeen AB15 8QH, UK; dan.fisher@glasgow.ac.uk (D.F.); margaret.currie@hutton.ac.uk (M.C.); kathryn.colley@hutton.ac.uk (K.C.); 2 School of Education, University of Glasgow, Glasgow G3 6NH, UK; 3 Environmental Psychology Research Group, School of Psychology, University of Surrey, Guildford GU2 7XH, UK; m.marselle@surrey.ac.uk; 4 Department of Family Medicine, University of Michigan, Ann Arbor, MI 48104, USA; swarber@umich.edu; 5 Nova Institute for Health, Baltimore, MD 21231, USA

**Keywords:** outdoor walking, nature-based intervention, wellbeing, social health, social wellbeing, loneliness, social support, group cohesion, social environment

## Abstract

Physical distancing practices during the COVID-19 global pandemic contributed to a high degree of social isolation among older adults. To reduce loneliness and other ill effects of social isolation, public health experts recommended outdoor social gathering, with physical distancing. Adopting a case study approach, we explored how social aspects of group outdoor health walks (GOHWs) mitigate social isolation for older adults and improve individual social wellbeing. We used semi-structured interviews to understand the experiences of social isolation and social relationships in nine older (50–80 s) adults participating in a GOHW in Scotland, United Kingdom (UK). Verbatim transcripts were analysed through an iterative process of thematic analysis carried out by an interdisciplinary team of qualitative researchers from environmental psychology, medicine, and geography. Themes provide insight into the social dimensions of GOHWs, the mediating effects of social experiences, and the contribution these make to individual social wellbeing. GOHWs provide opportunities to be part of a group and attend to the needs of inexperienced or physically challenged individuals. Being part of the group walk fosters casual interpersonal interactions through spontaneous mixing during and after the walk. This programmatic structure counters loneliness, engenders pleasurable anticipation of regular contact with others, supports physical activity, and fosters group cohesion. These in turn contribute to individual social wellbeing, including expanding social networks, meaningful relationships, a sense of belonging, and acting on empathy for others. GOWHs may be beneficial for mitigation of social isolation as we emerge from the COVID-19 pandemic. Findings were used to propose a conceptual model to parse social constructs and inform selection or development of quantitative social measures for future studies of nature-based interventions such as GOHWs.

## 1. Introduction

The COVID-19 global pandemic has required humanity to practice physical distancing as a way to mitigate spread of the virus. This has brought with it a degree of social isolation and increased loneliness experienced across demographics, but especially affecting older adults [1,2]. To reduce such effects, public health experts recommend outdoor social gathering (with physical distancing), identifying a need for evidence about interventions that could best address social isolation in older people and highlight the importance of “supporting… community use of local open spaces” [2] (p. 1163). In this paper, we present a qualitative case study exploration of older adults’ experiences of the social aspects of group outdoor health walks (GOHWs), undertaken prior to the implementation of physical and social distancing measures to reduce the spread of COVID-19. From this analysis, we draw out the interplay of social constructs in group nature-based interventions (NBIs) that might mitigate social isolation and improve individual social wellbeing for older adults. We use this insight to propose a conceptual model and offer recommendations for further study and implications for practice.

### 1.1. Risks of Social Isolation for Older Adults

As someone ages, their chances of living with a long-term illness, living alone, and being housebound increase. As one grows older, the number of people in an individual’s social network decreases [3]; this is particularly true for the oldest old [4]. Consequently, older adults can find it more challenging than other age groups to get out of their homes, interact with other people, and engage with natural environments—all of which are activities that are known to promote wellbeing [5,6,7]. 

Social isolation has been described as a lack of (or limited) social contact. It is considered an objective condition of an individual’s situation [8,9,10] and described as a state when “an individual lacks a sense of belonging socially, lacks engagement with others, and has a minimal number of social contacts which are deficient in fulfilling quality relationships” [11] (p. 9). Loneliness is generally seen as a separate yet related phenomenon. It is a subjective assessment [8,9,10], “based on a person’s emotional perception of the number and/or quality of social connections needed in comparison to what is being experienced at the time” [11] (p. 9). 

A recent consensus report suggests there is robust evidence that social isolation significantly increases the risk for premature mortality, with some evidence suggestive that the magnitude is “comparable to or greater than other well-established risk factors such as smoking, obesity, and physical inactivity” [9] (p. 42). In a study examining whether the association between social isolation and mortality was mediated by loneliness, Steptoe et al. [12] found that mortality was higher among more socially isolated and lonely individuals. Social isolation and loneliness have been found to be associated with cardiovascular disease, decreased physical activity, and decreased cognitive function [13]. 

Evidence emerging from initial ‘stay-at-home’ and subsequent phased re-opening COVID-19 mitigation measures implemented in many countries worldwide (e.g., 23 March to 29 May 2020 in Scotland, UK) highlights the effect of these on loneliness. In older people within the general population and specific sub-groups (e.g., living in long-term care facilities), loneliness levels increased [2,14]. Additionally, the intersection of vulnerabilities within older people—for example, their pre-existing increased risk of loneliness as compared to other groups and increased risk of serious illness or death from COVID-19 worsened by underlying health conditions—meant that this population sub-group were disproportionately affected by social isolation as a result of stay-at-home orders and social distancing measures [15,16,17].

### 1.2. Interventions to Mitigate Social Isolation in Older Adults

While individual experiences of ageing are not homogeneous, addressing social isolation and loneliness is an identified public health issue [18]. Within the UK, loneliness is an established health priority [19,20] and the World Health Organization (WHO) specifically lists social support networks as a determinant of health [21]. Maintaining social networks and participating in activities that promote social interaction are recognised as important for both health and wellbeing in older adults [22,23]. Systematic reviews have examined the effectiveness of interventions designed to alleviate social isolation and loneliness in older people [11]. The most effective health promotion interventions on social isolation and loneliness among older people tend to be group-based social activity interventions that target specific population groups [8,24].

### 1.3. Group Outdoor Health Walks

There is growing interest in the social health promoting effects of the natural environment [25,26,27,28]. Finding ways to combine access to natural environments to facilitate social interaction are supported by a number of international ageing strategies aiming to promote health and enhance quality of life during ageing [29,30]. Consequently, one potential intervention for social isolation is group walks in nearby nature. 

Walking is a behaviour that is influenced by the physical (both natural and built) and the social environment [31]. People are more likely to walk in the company of another person [32] and prefer walking outdoors with others more than walking outdoors alone [33,34] as it may help diminish any perceived danger of being alone [35]. As such, one way to increase walking behaviours is to utilise interventions targeting these two environmental determinants—physical and social—in tandem [36], for example, through GOHWs. 

GOHWs are an NBI that seek to promote wellbeing or prevent chronic health conditions through walking within a group in a natural environment [37]. GOHWs are defined as a “short, safe, social, local, low level, led walk” [38] that lasts for no more than an hour. These non-health service interventions are typically run by third-sector or community agencies with locally based, trained volunteer walk leaders. The target population for such walks are individuals who are relatively inactive and would benefit from being more physically active. The majority of participants in GOHWs are older people [39]. 

GOHWs have been identified as providing a socially supportive setting that attracts people to commence and maintain participation in physical activity [40,41,42] and are advocated as part of the community-wide approaches to promote walking [43]. Irvine et al. [40] found that social networks—word of mouth from friends and family already in the GOHW—were more successful at recruiting new walkers than signposting from medical professionals. Outdoor group walk programmes also have high retention rates [44]. The social aspects of being part of a group, enjoying the company, or helping others were found to be one of the most important factors for participants’ retention in a GOHW [40].

GOHWs could therefore reduce social isolation and loneliness. However, despite considerable research into the physical and mental health effects of walking, there is little research into the social effects of group walks. Social isolation and loneliness are one of the most under-researched assessed outcomes [45]. 

Quantitative investigations of individual social wellbeing outcomes from walking show inconsistent outcomes. Kelly et al.’s [45] scoping review of quantitative studies examining walking and mental health found that walking behaviour reduced social isolation in one study [46], but other studies showed mixed evidence [47,48,49,50]. Similar inconsistent results with quantitative measures for social dimensions of health are also found in studies on GOHW. While Irvine et al. [40] found GOHWs significantly increased feeling “open to others”, other studies have found nonsignificant effects on quantitative measures for social support [39,51,52,53]. This is in contrast to qualitative research, which has consistently identified social benefits to participation in GOHWs (e.g., [54,55,56]).

### 1.4. Challenges of Measuring the Social Dimensions in Nature-Health Research

The pattern of results for social health outcomes from GOHWs—wherein qualitative research shows the clear presence of social benefits but mixed results in quantitative studies—suggests the challenge of measuring social dimensions of health and wellbeing. For example, while not exclusively a GOHW, Warber et al.’s [57] study on the wellbeing effects from nature-based camp experiences found no significant pre–post change in participants on the quantitative assessment of individual social wellbeing. Yet, qualitative results showed otherwise. Campers ranked the experience of ‘spending time with friends’ to be the most enjoyable and influential activity to their nature-based camp experience [57]. Qualitative interviews with campers clearly emphasised the social environment (i.e., the process of making friends, the importance of being part of a group), as well as how the physical wilderness environment facilitated interpersonal connection [57]. Future studies investigating individual social health outcomes in nature and health studies may need to use new quantitative measures for social health grounded in the understandings revealed in qualitative research e.g., [40,57].

### 1.5. Conceptualising Social Dimensions of GOHWs and Their Effect on Health-Negotiating Cross-Disciplinary Discourse

Given the measurement conundrum identified above and the lack of consensus regarding the definitions of core constructs related to the social environment [31,45,58], particularly in relation to NBIs such as GOHWs, it is worthwhile to review social concepts across studies informed by health and environmental psychology disciplines. Important constructs called upon to explain the relationship of nature and NBIs with health include social support [40], social capital [25], social cohesion [25], group cohesion [59] and social wellbeing [28,40]. In order to develop a better understanding of how programmatic NBIs, particularly GOHWs, might promote and improve individual social health and wellbeing, we briefly look at each of these constructs in turn.

#### 1.5.1. Social Support

Social support is a dominant construct within the health disciplines [60], particularly in public health [61], and forms part of Engel’s [62] biopsychosocial model of health which underpins much of medicine. A simple description of social support used in multinational studies includes having a primary support group, experiencing interest and concern shown by others, and the relative ease of obtaining practical help [63]. Eight items from the Duke Functional Social Support Questionnaire were recently incorporated into a new Biopsychosocial–spiritual Inventory Scale and further delineate the makeup of social support. These items covered being able to: talk to someone about problems related to self, family, work or financial resources; obtain advice on important matters; receive help when sick; receive love and affection; have others who care about you; and receive invitations to go out/be with others [64].

Adequate perceived or received social support correlates with reductions in morbidity and mortality and is associated with better physical and psychological wellbeing and more life satisfaction [60]. Generally, social support buffers individuals from the negative effects of stress on both physical and mental health [65,66].

#### 1.5.2. Social Capital

Social capital may be defined as “connections among individuals—social networks and the norms of reciprocity and trustworthiness that arise from them” [67] (p. 19). Although definitions of social capital are contested [68], it may broadly be conceptualised as having an individual-level component as well as applying at the societal level [69]. Hartig et al. [25] highlight the role of contact with nature on the personal dimension of social capital, defining it as referring to “those resources available to an individual through his or her social connections, which may be activated in times of need” (p. 215), a definition reminiscent of social support. Neighbourhood greenspace in particular may provide a setting that fosters social capital formation. Domains of social capital can include empowerment, participation, group activity and common purpose, supporting networks and reciprocity, collective norms and values, trust, safety, and belonging [70]. The perspectives on social capital described here rely heavily on social networks that are also an identified driver of social support provision. When social networks are inadequate, the risk for loneliness and isolation increases.

#### 1.5.3. Social Cohesion

Hartig et al. [25] and Marselle et al. [28] favour social cohesion over social capital conceptually as a mediator in models of greenspace/nature effects on health and wellbeing, drawing on the following definition: “shared norms and values, the existence of positive and friendly relationships, and feelings of being accepted and belonging” [25] (p. 215). Social cohesion domains can include social networks and social capital as well as: common values and civic culture; social order and social control (including respect for differences); social solidarity; less wealth disparity (including willingness to help others); and place attachment and identity [70]. Biodiversity and availability of greenspace or other natural spaces at the neighbourhood level affects health, particularly social health of residents and users, by providing environments for positive interactions. However, research on this possible mediation is mixed and still in its infancy [28].

#### 1.5.4. Group Cohesion

Closely related to social cohesion, but focused on the group, not societal level, is the concept of group cohesion. This is defined as a dynamic process between the forces to remain in a group and resistance of the group to disruptive forces [59,71]. The forces to remain part of the group revolve around the attraction to the group goals and the group’s ability to mediate the goals for the member [59]. 

Carron et al. [71] developed the Physical Activity Group Environmental Questionnaire, which measures the extent to which the exercising group satisfies the participant’s needs and objectives, and the closeness and bonding of the group. Using this measure, group cohesiveness was associated with individual adherence to group exercise classes, recreational sports, and elite sports [71]. The questionnaire was also used in a study on an outdoor group walking program in Australia, ‘Just Walk It’ [59]. Example items were “Just Walk It is an important social unit for me”, “Members of our group often socialise during walking”, and “We spend time socialising with each other before and after walking”. Group cohesion was the sole predictor of adherence to the walking groups and was associated with a positive attitude towards physical activity [59]. Group cohesion appears to be critical to achieving health benefits from various group interventions, including NBIs that aspire to promote physical activity.

#### 1.5.5. Social Wellbeing

Individual social wellbeing, along with mental and physical wellbeing, is an integral part of the WHO definition of health [72], which has been incorporated into the most recent framework for studying the relationship of the natural environment to health, specifically its biodiversity [28]. Others [40,73] studying nature and health have advocated for using the biopsychosocial–spiritual model of health which also includes an individual’s social wellbeing as a core component [62,64,74,75]. 

Wellbeing stems from positive experiences, aspects, and evaluations across the domains of health, family, work, and economic status. It contrasts with the oft-measured quality of life which focuses on how deficits from illness, symptoms, injury, or pain affect these same or similar domains of life [76]. Perceived wellness depends on a multi-dimensional, salutogenic, systems view of life and includes social wellness, i.e., the support from family or friends as needed and the perception of being a valuable support provider for others [60]. Overall wellbeing is sometimes expressed as subjective wellbeing since it is a person’s subjective assessment of their own state of wellbeing. Keyes and Waterman [77] identify social determinants of subjective wellbeing as including social relationships, marriage, friendships, and social roles. In planning a new scale of subjective wellbeing, Lui and Fernando [78] note that social cohesion, belongingness, and support have been underassessed in previous work. 

After collating 99 measures for wellbeing, Linton et al. [79] suggest the following definition of individual social wellbeing: “concerns how well an individual is connected to others in their local and wider social community. This includes social interactions, the depth of key relationships and the availability of social support” (p. 12). Looking across three wellbeing scales that include subscales or items assessing individual social wellbeing, they each emphasise time spent with family and friends, including communicability (people to talk to) and protection (feeling safe and able to rely on help from others). Another major construct across these scales is the ability to help others, make a difference in their lives, and feel appreciated or loved by others [76,78,80]. A single scale includes an item on loneliness [80].

### 1.6. Conceptual Model for Investigating NBIs

To conceptualise or measure key social influences and social health outcomes of NBIs, such as GOHWs [31], a clear conceptual model is needed. Such a model could facilitate development of targeted interventions and generate evidence that will influence health practitioners, public health officials, and health funders [81]. GOHWs (and other interventions to reduce social isolation and/or loneliness [11]), are inherently complex as they have several interacting components (i.e., the activity, natural environment, the social environment and the delivery, personnel, resources, and fidelity of the programme), which additionally interact with characteristics of the individuals involved (e.g., age profile, health status, motivation) [82]. All these components and characteristics need to be sufficiently described in order to identify which aspects of an intervention are most effective for which specific population and in which particular context [11].

**Figure 1:**
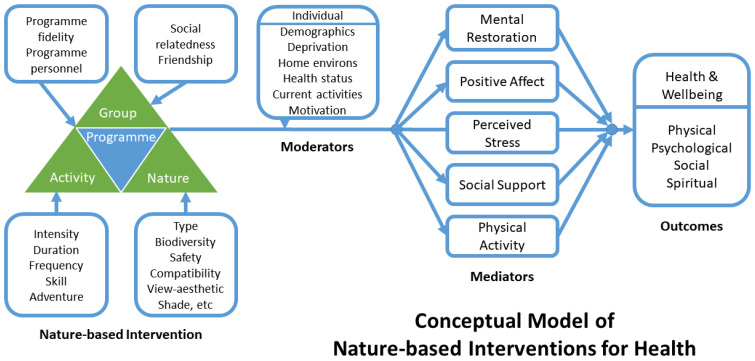
Conceptual model for investigating nature-based interventions that aim to promote health through behaviour change. The model illustrates aspects of the programme, mediators and downstream health and wellbeing outcomes. Associations between variables are subject to modification by individual characteristics (e.g., demographics). (Modified from [40]).

The Irvine et al. [40] conceptual model specifies the aspects of NBIs that aim to promote health through behaviour change (Figure 1). The model identifies four components of a nature-based complex intervention, such as GOHWs: the activity of walking, the influence of nature, the effects of being part of a group, and programme delivery effects. The model incorporates attributes of the individual and their life context that potentially modify the associations between features of the NBI and outcomes. The model additionally details potential mediating pathways through which an NBI might affect health and wellbeing. Health and wellbeing outcomes are specified according to the biopsychosocial–spiritual model of health [40,73]. Noticeably, social dimensions are located in three areas: (i) the group aspect of the programme (social relatedness, friendship); (ii) a possible mediator of effects (social support); (iii) and as a health and wellbeing outcome (individual social wellbeing, loneliness).

### 1.7. Study Focus

One conundrum in nature–health research is how social dimensions of groups, the physical elements of nature, and various types of activity drive or possibly confound the documented effects of NBIs on health and wellbeing. A clearer understanding of the social dimensions of NBIs, such as GOHWs, is needed to disentangle these issues and to understand the potential for such programmes to mitigate social isolation and loneliness. 

The aim of this study is to qualitatively explore older adults’ experiences of social isolation and social relationships in the context of participation in GOHWs. Such an inquiry can inform our understanding of the interplay of social processes and dimensions in outdoor group walks and guide the development of social-focused measures for use in research of NBIs. The specific research questions include:

1. How do individuals taking part in an NBI for the promotion of physical activity articulate the social processes and outcomes they experience?
2. What are the salient dimensions of the social environment related to outdoor group health walks?
3. How can the conceptual model of NBIs for health be adapted to illustrate the social dimensions for individual social wellbeing?

## 2. Methods

### 2.1. Research Design

We employed an instrumental case study design using qualitative methods [83] to enable a group of people, through their individual stories, to explain their experiences of participation in a GOHW in rural Scotland, UK. This case study approach offered a more flexible analytic process than, for example, grounded theory or phenomenology [84] and addressed calls for the use of qualitative methods for giving clearer insights into the experiences of older people and their engagement with natural settings [85]. We incorporate both description and interpretation [86], thereby providing nuanced “thick descriptions” [87,88] of older people’s experience and interpretation through the development of a conceptual model that seeks to illustrate how emerging experiences are interlinked [89] to inform future research.

### 2.2. Recruitment Process

The Cairngorms National Park, located in Scotland, UK, as part of their Active Cairngorms strategy which aims to enable use of the park for physical activity, facilitated a 12-week GOHW (July to October 2017) using an activity tracker to document individuals’ physical activity. This was the second such activity tracker for GOHW undertaken to specifically promote physical activity amongst individuals who live within the national park and to engage local doctors’ surgeries to ‘signpost’ individuals to the walks. Recruitment was additionally carried out through promotional flyers posted on notice boards in the target community, including the doctor’s surgery, and distributed door-to-door (see [40] for results of a mixed-method study on the first walk).

Eleven individuals completed the second 12-week GOHW activity tracker. The group continued walking beyond the end of the 12-weeks, with several individuals undertaking training to become volunteer walk leaders for the group. All walkers were advised of the current study opportunity by the Cairngorms Walk Leader. At the invitation of the walkers, one of the researchers (KNI) joined a walk and the after-walk coffee gathering as an opportunity to meet individuals, further discuss details of the research (e.g., purpose, how data would be used), and gain first-hand experience of the walk. All 11 individuals expressed interest; 8 took part in the current study. Non-participation was due to ill health. An additional individual who had joined the GOHW activity tracker after it started and never used an activity tracker also participated in this study.

Ethical approval was granted by the James Hutton Institute’s Research Ethics Committee (119-2017).

### 2.3. Participants

The nine participants all lived within the same locality, a village located within the Cairngorms National Park within Scotland, UK. Participant ages ranged from 50–80 plus years. Two were widowed, one was single; there were three couples.

### 2.4. Data Collection

Data were collected through seven individual and one paired (P6 and P7) semi-structured interviews for a total of eight interviews with nine individuals. The interviews took place in April 2018 and were conducted by one researcher (KNI). Following a reminder of the study’s focus, an assurance of our interest in hearing their thoughts, an explanation of confidentiality and anonymity procedures, and obtaining written consent, interviews were conducted in-person at participants’ own homes or a mutually convenient location (e.g., local coffee shop); when interviews took place in a public setting, we sought to ensure privacy by arranging the table so that it was out of earshot from others. The interview schedule included open-ended questions and prompts broadly focusing on the facilitated 12-week GOHW activity tracker followed by their reflections on the experience and its effect after 6 months. 

All interviews started with a question asking participants to share a story about a specific walk or part of the 12-week GOHW that was special to them. One set of questions explored recruitment-related topics, including their motivation to join, how they got involved, and any barriers to joining. A second set sought their reflections on the implementation of the 12-week GOHW activity tracker and the experience of using the activity tracker. The third set asked about the walk locations and their experience of those locations, including being out in nature. Before moving to the closing set of questions, participants were asked to share anything else about the 12-week walk that was important to them.

The last set of questions asked participants to reflect on the experience and its influence on their lives 6-months later. Prompts suggested that they might consider this in terms of time spent outdoors, physical activity, feelings about themselves, and their friendships. A final closing question offered participants the chance to add anything else they would like to share. Interviews took approximately 50 min (range 26–80 min).

### 2.5. Analysis

All interviews were audio-recorded, anonymised, and transcribed verbatim by an external transcription service. The interview transcripts were analysed through a process of in-depth thematic analysis [90], carried out by a team of four interdisciplinary researchers (environmental psychology, medicine, and geography). NVivo (version 12) was used to create an initial coding frame based on the interview questions, which had been developed by drawing on the existing GOHW literature (e.g., [39,40] model for investigating GOHWs as an NBI). Further codes were added as these emerged in the analysis. We thus incorporated both a researcher-anticipated (etic) and participant-derived (emic) approach to data analysis [91]. The emic approach was supported by an iterative team process that allowed key themes to emerge from the data. 

The process began with the coding of one interview by two researchers (KNI, MC), development of consensus, and application of resultant codes to the remaining transcripts independently (KNI, MC, DF). Data were initially analysed using the etic theme of social interaction. Next, emerging emic themes focusing on the group aspects of the programme’s design, social processes occurring on the walks, and resulting individual social wellbeing outcomes were subsequently organised using Irvine et al.’s [40] conceptual model as a frame. The data were then systematically analysed a third time by applying the newly defined emergent themes across the full interview material (KNI, SLW, DF). 

## 3. Results

In this section, we detail the social dimensions of GOHWs using four themes: programmatic elements fostering engagement; spontaneous mixing and mingling; evolving social experiences; and achieving individual social wellbeing. These and their related sub-themes are summarised in Table 1 and described in turn utilising quotes from participants to offer a rich illustration of findings. The descriptions purposefully incorporate both short and extended quotes from multiple participants to: (i) provide sufficient contextual information concerning the development of the group’s social dynamics and the subsequent effects on individual social wellbeing of group members; and (ii) facilitate transparency for subsequent interpretation of findings into a conceptual model and recommendations for future study.

### 3.1. Programmatic Elements Fostering Engagement

The programme provides the opportunity to be part of a group and a structure that attends to the needs of inexperienced or physically challenged individuals. The following quote exemplifies the importance of this dual value for people who joined up:

Particularly people who are getting on in life sometimes have difficulty fitting in because they’re older, because they feel ‘I don’t really know these people’, and it’s very important for their wellbeing if they’re in a group with people of similar problems, … I don’t think some of the folk have done an awful lot of walking and I think they need a bit of assistance. … if they felt that they couldn’t do the full walk then there was the freedom to stop and come back, and somebody would go with you. So, there was an element of security, I felt, as far as they were concerned that somebody was going to look after them and accompany them… (P8)

First, we focus on elements and processes of the opportunity to be part of a group. Some people were drawn into the walking group by existing social connections with others who knew about the group forming. Signposting at the local surgery (general practitioner’s office) was another way walkers found out about the walking group. Others came to the group specifically “… hop[ing] that it would increase the social side of things. Seeing other people” (P3). One walker remarked, “It brings people together and get to know each other that probably have totally different interests in other ways and probably wouldn’t have [otherwise]” (P9).

One person described their first visit to the walking group remarking on the welcome they felt, despite not knowing anyone:

There was a couple of the golden oldies of the village in there, so I recognised their faces, so that was good. There was [the walk leader’s] beaming smile and, ‘Hello, come in!’. … [And] everybody else in the group made me feel really welcome the first day. (P1)

For this individual, the walk leader was instrumental in setting a welcoming tone. A testament to the overall success of the group opportunity was that “… new people have joined the group which is an indication that we’re all quite enthusiastic about it because people have said, ‘Oh, why don’t you join the group?’, and they have done. … [and] … they’ve all stayed…” (P6 and P7). 

The second part of the success of the group walking programme was the structure that could support varying needs, including training of walk leaders, documentation of group members’ health needs, assessment of walks, and member responsibility to report on their immediate needs. As an example, for this individual, the walk leader’s role in accompanying and encouraging a slower walker made all the difference for their involvement:

A few times, I did go with the group, but I’d used to have to have a seat and they’d go, ‘Bye’ and I’d sit there and wait for them but with [the new walk leader], I didn’t do that. … He made you want to walk. I mean, I remember my first day with him and I was at the very back, he was at the very back with me. … he said, ‘No, you’re not walking on your own’, and he was just so good. (P6 and P7)

People with diverse abilities and challenges made up the group, including older people, “… they’re all sort of 80 s … [and] this elderly couple, he’s coming onto 90 … It’s good that we can go at a pace for everybody” (P2). Matching the walking location with the abilities is also a key to success: “up there … there are possibilities for everybody to be catered for and do slightly different walks” (P9). Finally, assuring the safety of everyone makes it all possible: “… some people are out walking and … they collapse. It’s reassuring if you’ve got somebody with you who can get you home or to the doctor or call the ambulance. There’s a feeling of security in that, I think” (P8).

### 3.2. Spontaneous Mixing and Mingling

Being part of the group walk fosters casual interpersonal interactions through spontaneous mixing during and after the walk. Participants described how during the walk, the regular breaks or small hurdles that needed to be navigated while out walking (such as slippery areas or roads to be crossed) were conducive to fostering group interaction and changes in walking partners. As one walker explained: “you’re… chatting to somebody one minute, you cross the road, they’ve gone there, then you’re away to somebody else. It just seems to flow, it’s really nice” (P1).

This spontaneous ebb and flow of movement was further described as a “mov[ing] backwards and forwards” (P2). This walker detailed the way in which one’s placement within the group at any one time shaped these interpersonal interactions:

I think it’s just strolling along on the walk… the group at the front… they were all … talking to each other. I was at the back…and we are talking… about this, that and the other, so there is a bit of social intercourse there. (P2)

The different seasons and environment in which the walks took place, the step count activity trackers in use by many of the walkers and the occurrence of local or national events also sparked spontaneous interaction. One participant spoke about these conversation catalysts as: “You’re going at different times of the year so you’re seeing different things coming up… We peek over peoples’ garden fences and we start talking about their plants and that gets them going about their gardening” (P1). An additional walker noted:

We’ve all got our own individual [activity tracker] that we look at, and because we’re doing it individually, we are discussing it as a group as well. Some people would turn around and say, ‘Well, I’ve done more steps than you.’ It didn’t become a competition or anything like that… It was a recognition that you got by having a tracker. (P3)

Not everyone wanted to do their talking on the walks. For one this was due to the effort noting that “I don’t mean to be rude but I don’t do much talking … because I’m saving everything [for the walking]” (P3). Taking an interest in the walk environment was another reason, as described by this walker: “[I] don’t really want to talk too much … because I like to look for the birds and the scenery and everything” (P9). Their preferences were to enjoy their talking afterwards.

Gathering after the walk provided additional opportunity for casual interaction and was considered “just as important as the walk” (P4). The preparation (or purchase) of a hot beverage to have together generated laughter and to “talk about something in the village, the local scandal or whatever it might be” (P8). One individual noted how “I like the banter afterwards… because you don’t always get to speak to everybody on the walk” (P1). Participants observed that this after-walk socialising would sometimes take place even if a walk was cancelled due to poor weather; “We’re not stupid about it… if the weather [is] atrocious we just have a coffee and the après walk” (P3). Some individuals would also “go later for the coffee” (P6 and P7) if they were unable to join the walk for health reasons.

Participants generally noted how the walking outdoors in a group and the after-walk socialising enabled people who otherwise lead quite different lives to come together and informally enjoy a combined physical and social activity. One walker described this as “you’re chattering away…and finding out different ways of life that people have” (P5). For another walker, being in a walking group provided a different experience, allowing them to get to know people in a more casual way:

It’s just the being in a group rather than walking [by] yourself or with a friend… you don’t compare [the walking group] to coffee mornings because [they are] a bit static, where you go for coffee somewhere so you feel you’ve got to invite them back. [Instead] you can go for a walk with the group and it’s a casual friendship getting to know people, which appeals to me. (P9)

### 3.3. Evolving Social Experiences

The spontaneous socialising supported by the ‘programmatic elements’ theme and the ‘mixing and mingling’ theme set the stage for largely positive and evolving social experiences. Four sub-themes were identified within this third theme. These include counteracting loneliness, anticipating regular contact, supportive socialising, and emerging group cohesion.

#### 3.3.1. Counteracting Loneliness

Several participants identified combating loneliness or social isolation as a reason why the group walks were good for people’s wellbeing. They spoke eloquently of their own personal loneliness, their knowledge of others’ isolation, and the ways in which the walking group had made a difference for themselves or others. One individual explained it like this:

I would reckon that the social implications of anything you do like… walking for health, the benefit is a 60/40 in favour of the social side. It might be 70/30… it’s certainly, in my opinion, more important than the actual activity itself. I thought… this could be of some use to people … because a lot of people are… lonely. (P8)

Living alone was a set up for a sense of isolation and a self-identified need to join the group walking programme. Two participants were particularly forthcoming about their situation:

My dad passed away so I was on my own. I’m not married and I don’t have children. I was kind of lost after dad passed away. … and I was like, ‘What do I do with my life? What is this?’, and my mood was dropping a bit. I don’t know if it was the grieving process or what. [The walking group provided] company for me because I’m on my own, I’m in the house. … if you don’t see the neighbours then you could go all day without anybody. (P1)

I’ve been a hillwalker for 70 years. … I like walking and being out. … Living alone, I thought, ‘Well, this is good because other people with similar sort of things might join up…’. (P8)

Other reasons for isolation included a marriage in which the children were gone, the wife was depressed, and the husband isolated himself in his “man shed” (P4). Another joined because she did not know people in the area (P6 and P7). The walking group helped these individual situations because of “meeting new people in the village” (P1) and “giving … back confidence” (P1). The programme was an opportunity to “take us out and do something with other people” (P8). 

#### 3.3.2. Anticipating Regular Contact

The weekly walk provided an opportunity for regular contact with other people, breaking up routine, and something to look forward to. Participants spoke of making a point of attending the weekly group walks. One individual somewhat proudly noted “We’ve been every Friday now. We haven’t missed once since last July” (P5) while another commented that: “People just got used to the idea that on a Friday, we’re going walking… ’are you going on a walk today?’ … was the favourite question” (P8).

These opportunities for weekly contact were anticipated with pleasure as illustrated by comments such as “Oh, it’s Friday today, walking group; [don’t] want to miss it” (P4) and “it’s something to look forward to. I do enjoy meeting up on a Friday” (P1). For one participant who suffered from depression and had been encouraged by their doctor to get out more, they described a shift in their feelings toward the weekly walk from trepidation to enjoyment: “Certainly for the first couple of weeks I felt that I had to push myself to go, but then seeing people there, that quickly subsided to be taken over by ‘hey, I’m looking forward to going’” (P3).

#### 3.3.3. Supportive Socialising

Supportive socialising refers to the chatting and the camaraderie that helped people join and complete walks they would not have attempted on their own. One person commented, “the social side motivated you into actually doing [the walks]” (P6 and P7). Their spouse with respiratory issues concurred, “I wouldn’t be on those walks for any other reason but I joined the walking group” (P6 and P7).

Others specifically identified the chatting with each other as the type of support that helped them during the walks. For example, “You don’t notice how far you’re going because you’re having a chat. It doesn’t seem to be an effort. … The other day I walked to here, which I would have never dreamt of doing last year” (P2). Another described how a walker was so involved in speaking to everyone else during the walk that “The time just flew past and they didn’t realise they’d gone that far” (P4). 

One person acknowledged that, “People always talk except if it’s a rather strenuous walk then you can’t talk” (P8). That is where the general camaraderie of walking with others may have become the more important aspect of support. One participant described how pain could be overcome:

When you’re out walking with people and the other men, the camaraderie of everybody and socially, you forget all about that. You just get on with it. … Yes, you don’t sort of dwell on yourself. You get out there and do it which is good. (P4)

One of the couples in the group reflected on a time when the more physically restricted of them walked up to see a viewpoint where the others were, rather than just get in the car alone and wait (P6 and P7). They also commented about the encouraging effects of the group in general:

P6: If we did [the walk] on our own, we probably would have turned back and finished it earlier. This is where the group…

P7: The group comes in, yes. I would have turned back, I would have definitely turned back but because there were other people there, I was … encouraged. (P6 and P7)

Another person noted discovering that they could push themselves a bit further on walks they undertook with a friend (P3). This same individual went on to describe how the supportive socialising of enjoying being together built confidence in the group members’ abilities:

I think it was getting everybody together and realising that we can go further and that it’s enjoyable together. So, there is the motivation, there is the social side, and there is the improvement in your health and ability. Thinking back to the beginning of it, and then as we went through, I’m pretty sure that the majority of people would be comfortable … [and] confident in their ability to go further. (P3)

#### 3.3.4. Emerging Group Cohesion

Participants reflected on the emerging unity and cohesion within the group in the context of decisions about the walking. Dynamics of decision making could also be a potential disruptor, along with new people joining. Decisions were frequently described as collectively made. An initial key decision was whether to carry on walking after the organised 12-week GOHW activity tracker ended. A participant described this process as “there was a brief discussion and it was agreed that we should continue as a walking group” (P8). Additional ongoing task-focused decisions included whether to continue to utilise the activity trackers, whether to organise walks that required transport by car, and the location for after-walk socialising over coffee and tea. One participant’s description of the process for this last decision is suggestive of the growing cohesion and desire to make it simple for people to get together:

[Some walkers] thought we should patronise different [cafés] … We did try that, but there was a problem in that if you missed a week, where are we going to meet? Eventually, we got everybody to agree that we should stay at one location so that everybody knows, ‘This is where we meet, if at all possible’. (P3)

Walk locations were initially selected each week at the start of the walk through a process whereby individuals would put forward ideas with the final decision made based on “majority or we just go with the flow” (P1). This decision process was itself a point of discussion which resulted in a different approach, as described in the following comment:

We’ve had discussions … and there has been a suggestion that we pick two walks each … and perhaps prepare beforehand… I think we’re probably going to settle for thinking about two walks each, two within the village and two out with the village. (P3)

Potential disruptors of group cohesion were also raised. These included inability to involve everyone in the group decision making, arguments about walk length and the addition of new people. In reflecting on the group’s decision making, an interviewee observed occasional reluctance to discuss where to walk and what the group was hoping to achieve:

There’s been a bit of reluctance sometimes within the group to discuss things and hear different people’s opinion … People tend to go off and speak quietly to each other … about the walks … about the group and what we’re aiming for … but not bringing [this to the group] … [to] hear different people’s ideas. (P9)

Arguments over whether the group would stay together while on a walk or whether to shorten a walk were another potential disruptor to group cohesion, illustrated as follows: “… sometimes you suggest a walk and the other people don’t agree, they want to go somewhere else or they want to shorten it. They want to shorten it because they don’t want to walk that far” (P4). To reduce this tension—and increase group cohesion—it was suggested that all group walkers should attend a walk leader training course, even if not planning to take on a leadership role. This would, as one participant commented, generate “greater awareness of what is involved—safety and all sorts. If everybody did it, it means there would be no arguments” (P5).

Another potential disruption to group cohesion was associated with bringing new people into an existing group. While one participant observed that “new people have come and we have embraced them” (P2), another noted that some individuals were worried about how new people would fit in, because “You’ve got to enjoy the company of the people in the group.” (P3). The strength of the group’s way of interacting and doing things was noted as a potential buffer to this type of disruption:

I like to think that if we’d just carry on the way we are if somebody comes along that doesn’t fit in for whatever reason, that they’re not caring or they’re more dictatorial, it would just sort itself out. (P3)

Despite there being periodic areas of disagreement and some worries about new members joining, participants’ narratives highlighted group resolutions that increased cohesion over time. Their reflections also illustrated the strong motivations that individuals had to maintain and nurture the walking group going forward.

### 3.4. Achieving Individual Social Wellbeing

As a result of being part of the walking group, which enabled participants to exercise regularly and engage in meaningful social interactions with others in the community, our interviewees demonstrated increased personal social wellbeing by expanding social networks, making meaningful relationships, experiencing a sense of belonging, and acting on empathy.

#### 3.4.1. Expanding Social Networks

People with social wellbeing have a sense of expanding social networks and are learning about people in relation to the places around them. Each of these strands was present among our walkers.

In the first instance, people commented on how they were getting to know people in the area. Some knew a lot of people from their work or because of their outgoing nature, such as one who made it a point to get to know the most recent new person in the group (P9). Others, however, knew very few people at the beginning, such as the participant who wanted to “… know more people in the village, … even if it’s just, ‘Oh, hello’, or whatever, and it’s worked” (P1). Another who had lived in the area for 27 years hoped:

That there would be more village people involved in this; but local people indigenous to [the area], they’re sometimes reluctant. I think if you look at the people who are on this, you will find that it’s mainly incomers… [of less than seven years]. (P8)

In the second instance, participants felt comfortable further expanding their social network by liaising with other walking groups at some distance. For example, some walkers planned a trip to meet up with another walking group that was “quite a long way but it’s a lovely drive, so we’ll organise that and make sure that we’re there” (P4). At another time they all went and met other walking groups, “… we met several of them… It was interesting chatting with them about different areas” (P5). These growing social networks with other walking groups came in handy when someone was moving away: “she doesn’t know anybody, and I said ‘It doesn’t matter, there’s a walking group there and I’ll give her a name [of someone to contact]’” (P4).

The expanding networks also led to learning while walking about the local area and developing stronger relationships with these places. This was particularly relevant for past events as illustrated by the following comment:

People know the houses as well. You walk past the houses and, ‘Oh, such and such lives there’, and you wouldn’t know that before. Somebody would say, ‘Oh, I went to school there’, and it was the old school. … You get little stories about what happened. (P6 and P7)

Other times, it was the conversation topic after the walk over coffee or tea that expanded associations: “…when you’re in the [hall] and then they talk about things that are happening around the village which some people don’t know about” (P4). All in all, these expanding social networks and knowledge of the people and the places around them improved the walkers’ social wellbeing: “…we’re all different and we enjoy different things. I think the socialising side, … it’s definitely helped some people socially, probably even more than physically” (P9).

#### 3.4.2. Making Meaningful Relationships

One aspect of social wellbeing is to be able to make and maintain meaningful relationships and develop friendships. People in the walking group describe a movement from knowing people by sight to talking with them on walks to engaging with them in other situations and then realising that they had made friendships.

One individual spoke about how their impression of a person completely changed by getting to know them better:

Like this … old guy… I’ve always thought of [him] as quiet and he still is quiet but some of the comments he used to make … He was so tongue-in-cheek and he’s so funny. Now, coming into the shop or just meeting him in the street, you wouldn’t have known that but for sitting talking to him … He’s a font of all knowledge. (P6 and P7)

One walker described how the process of walking with others in a group facilitated a meaningful connection when they found themselves “going at the same speed” as a fellow walker, they discovered a mutual interest in the “… scenery and everything around, the environment around about” (P9). Subsequently, they had one of the longest conversations this individual had ever had with anyone in the group and they felt they really got to know the other person.

These types of interchanges underpin the growing friendships that were occurring. Two participants described how the walking group facilitated meaningful connections and the effect this had on their own feelings about themselves:

I’ve made friendships. A good laugh … you realise that it’s good to mix and not sit and dwell on things [like losses]. (P1)

While I knew some of the people on it, it has increased our friendship and camaraderie, I would say. I can be out … and I go past their house and there are waves … or if they’re out, we stop and have a chat for a few minutes and it’ll be, ‘I’ll see you Friday’. That actually makes you feel better in yourself. (P2)

The two individuals in the paired interview echoed the spilling over of these growing friendships to other situations outside the walking group. During the interview while describing how they were checking up on others who were unwell or that others checked in with them they suddenly realised “… so it’s actually formed sort of a friendship” (P6 and P7). The following comment summarises how relationships had changed within the group over time:

You probably know them as your neighbour and just wave, but now you know them a bit more personally than you knew before and you have more empathy towards them as well when you hear their life story. When you walk along, you always hear about what they’re doing or what’s happened to them. It’s very good both ways—walking and when you’re at the [hall] after you’re finished. It seems to have brought people closer together. (P4)

#### 3.4.3. Sense of Belonging

Another aspect of individual social wellbeing about which participants spoke was of having developed, over time, a sense of belonging to the group, including responsibility and loyalty. The group had “a sort of cosiness about it” (P9). This sense of belonging is expanded in the following comment:

I feel that the group is strong and we look forward to meeting up together, and I think that it has become caring and hopefully we can move on together by staying together. (P3)

This sense of belonging generated a feeling of belonging, loyalty, and responsibility. As one individual described:

You feel part of it and you want it to continue. I think that’s it. It’s a bit of loyalty to the group really. If we all said, ‘Oh, I can’t be arsed today’, it wouldn’t be a group, would it? (P6 and P7)

This sense of loyalty was also present amongst walkers who were currently inactive due to the winter weather. Described by one of the participants as “more fair-weather walkers [because] … they’re in their 80 s” (P1), these individuals had told the group of their intention to return to walking in the spring. Another situation described was that of a walker whose partner was unwell and who was uncertain whether to attend but did in the end: “He said, ‘I was tempted to just stay at home but no, I’ll just come out because I’ve got a group and I don’t want to let them down’” (P1).

#### 3.4.4. Acting on Empathy

Being part of the walking group resulted in a developing sense of respect and empathy for others’ physical ability and needs as well as individual differences, an important part of individual social wellbeing. Such awareness was taken into account when planning walks, for example, considering the terrain for and pace of walks as well as ensuring there were places to rest along the way (e.g., a bench for sitting)—and that everyone had an opportunity to take that break. 

Participants also spoke of actively looking after one another while walking. Examples include, the more fit walkers helping others up steep inclines or over rough terrain, and “everybody help[ing]” the individual “whose balance is not great” (P4). The following comment provides a rich description of how this respect and empathy is manifested:

There are some people with obvious problems and they’re getting through it their way, and that we do care about each other…. People don’t want to be a burden, but I think we can all realise that we’ve been through situations ourselves whereby we … [think] … ‘Hey, am I ever going to be able to do something like this again?’, and we can sympathise with people and help them through it. (P3)

Two individuals spoke of how their growing awareness of and respect for others’ needs motivated them to undertake training to become volunteer walk leaders for the group. One individual described this as follows: “It’s good to get out with some of the older people and help them along. That’s why we actually did the [walk] leader course” (P5).

An additional way in which participants spoke of the empathy and respect that had grown over the weeks of walking together was as an increased appreciation of different world views and ideas. One participant described the walks as “bring[ing] people together that probably have totally different interests” (P9). They went further to say:

For me, I’m just very happy if I see these different people enjoying themselves, getting on together, being sociable and being aware of each other and considerate to their needs without making a fuss. (P9)

## 4. Discussion

This paper reports findings from a qualitative case study exploration of older adults’ experience of social isolation and social relationships in the context of participation in GOHWs in Scotland, UK. Findings contribute to knowledge of the social dimensions of what are often characterised as NBIs to increase physical activity and promote physical health. In this way, our study responded to calls for further research into the social dimensions of the nature–health relationship, particularly in vulnerable populations such as older people [27]. Specifically, we identified salient dimensions of the social environment of GOHWs, the social processes, and the individual social wellbeing outcomes experienced by walkers. We offer a conceptual model through which to interpret the interlinkages of these social dimensions that might inform future research and programmatic initiatives to address loneliness.

Participants spoke of the enticement that the group structure of the programme provided for their motivation to be physically active and to increase the social side of their lives. The way in which the group structure was designed and implemented also was supportive for individuals with mixed abilities and experience. Additionally highlighted was the fluidity of spontaneous social interaction that occurred when on the walk. This mixing and mingling was facilitated by a natural ebb and flow of movement as people slowed or sped up their pace, stopped for a view, or attended to someone needing some assistance. The after-walk socialising added an additional casual space and opportunity for further unstructured interaction.

The programmatic group structure and opportunities for spontaneous socialising helped address some participants’ self-identified loneliness and social isolation. Conditions mentioned as contributing to either their own or others’ isolation included living alone, death of a partner, lack of physical fitness, being deaf or partially sighted, living with chronic illness, mental health issues, or displacement due to an environmental disaster (e.g., flooding). Participants highlighted having something to look forward to and the positive emotions associated with anticipating getting together on a regular basis. The supportive socialising offered through the group enabled walkers to walk further, overcome physical challenges, and achieve more than anticipated. They spoke of an emerging sense of a unified group generated through shared decision making and a shared commitment to walking. The potential for disruption of this group cohesion was highlighted with participants noting concern about possible financial barriers to joining and the potential impact that new people might bring to the current dynamics of the group’s workings. Participants’ comments suggested an increase in individual social wellbeing: they were expanding their social networks, making meaningful relationships, developing a strong sense of belonging, and acting on a growing empathy and respect for others.

### 4.1. Mitigating Social Isolation

The potential for GOHWs to mitigate social isolation and reduce loneliness is evident in our participants’ experiences. This finding contributes to a qualitative body of research that identifies social connection as an important part of group walks [55]. Taking part in a regular organised walking group reduces social isolation and provided an incentive to leave the house [41]—especially important for older adults who were bereaved [41,92]. Indeed, “social contact/reduced sense of social isolation” was one of the most commonly cited benefits of participation in a health walk in England, UK [54] (p. 32). As Rigby et al. [93] identify in their scoping review of walking groups that seek to promote physical activity and health, such groups “may present an opportunity for those feeling isolated and lonely” (p. 16). Our findings combined with those of others begin to provide good support for such a proposition. 

### 4.2. Social Elements of the Intervention

Our theme ‘programmatic elements fostering engagement’ explored how the programme structure facilitates group inclusion and supports walkers with diverse abilities. This enabling role has been found in studies on specialist walking groups created for people with long-term or short-term physical health needs, such as recovering from surgery [41,92], long-term unemployment [92], or mental health problems [55]. Walking groups made up of people who are similar can help create an inclusive “safe space” (p. 5), which can be crucial for people’s decision to join a walking group [92]. We provide new insight that suggests these programmatic elements can effectively engage groups of individuals with differing health and social needs as well. 

Our theme ‘spontaneous mixing and mingling’ identified how being part of the group walk offered a casual space and opportunity for socialising through the mixing, ebb and flow that occurred during and after the walk, a phenomenon referred to by others as “fleeting sociability” (Doughty, 2013, cited in [55] (p. 7)). This experience of spontaneous socialising during a group walk resonates with and further supports previous studies [54,55,92,94]. While our participants spoke mainly to these moments of sociability as taking place with other group walkers, such interaction has also been found to occur with passers-by external to the group [92]. The ebb and flow identified by our participants may enable both the development of interpersonal connections and the opportunity for individual experience building, which have been noted as important constructs within leisure studies [95]. 

### 4.3. Mediating Social Experiences

Our third set of themes ‘evolving social experiences’ encapsulated the evolving, largely positive, social experiences in GOHWs that can follow from the programmatic elements and the spontaneous social interactions identified above. Social experiences comprised four sub-themes which, as a whole, provide useful insight to filling a gap in understanding the “self in social contexts, the dynamics of social engagement” from nature-based leisure [95] (p. 618). The first two sub-themes, ‘counteracting loneliness’ and ‘anticipating regular contact’, illustrated how the social aspect of the GOHW helped address some participant’s self-noted loneliness and highlighted the positive anticipation and regularity of contact from the GOHW. These provide further qualitative evidence that such structured NBIs can support the development of new routines, especially for people who had lost a sense of rhythm to their days due to retirement or bereavement [54,55,92], and offer a way toward reducing social isolation and loneliness. 

The third and fourth sub-themes articulate experiences that differ from descriptions of findings in previous group walk research. ‘supportive socialising’, the third sub-theme emphasised the way in which talking while walking enabled walkers to achieve more walking than they anticipated being able to do. This differs from Dawson et al.’s [54] descriptions of social support in the walking group, which emphasised the availability of social resources to cope with life challenges outside of the group walking experience (e.g., a broken ankle). The ‘support’ discussed in our study is one that focuses on the processes and dynamics between individuals in the group while walking, identifying an additional possible salient dimension of the social environment associated with group walks.

Our final sub-theme ‘emerging group cohesion’ identified both positive and negative dynamics of group decision making and perceptions of how this emerging group cohesion could be affected when new people join. This is distinct from social cohesion as discussed in previous studies which identified how participation in group walks can lead to “‘spin-off’ activities” [41] (p. 23), such as meeting for coffee [41,92], informally organising their own walks outside of the main group [54,92], or trips out [41]. Rigby et al. [86] argue that social cohesion is especially important for addressing physical health inequalities in outdoor walking groups. In contrast, our study emphasises group cohesion, the bonding of a group with shared goals, previously found to predict adherence to a group exercising and group walking programmes in Australia [59] and the United States [96]. 

### 4.4. Individual Social Wellbeing as an Outcome

Our fourth, and final, theme ‘achieving individual social wellbeing’ highlighted four social wellbeing benefits walkers gain from participation in a GOHW. These benefits emerged from the previously described ‘evolving social experiences’ theme. The first three sub-themes lend further evidence to previous qualitative studies on walking groups as to the importance of these social processes [41,54,55,92,94]; here we interpret them as health outcomes, heeding calls to identify and parse longer term effects from leisure time [95]. Our first sub-theme ‘expanding social networks’ identified how participating in the GOHW expanded walkers’ social networks and knowledge about people in relation to the places around them. This is in line with South et al.’s [41] qualitative study in which walkers reported the development and strengthening of social networks and the potential for those to increase contacts in the local community. Interestingly, our participants also emphasised the relevance of place as part of those networks. ‘Making meaningful relationships’, the second sub-theme, highlights the ability to make and maintain meaningful relationships and develop friendships as important aspects of individual social wellbeing. Previous studies have described this “meeting people and forming friendships” as a cross-cutting theme in the social benefits of GOHWs [41] and clearly a “powerful experience of group walking” [55]. 

Our third sub-theme of ‘sense of belonging’ identified the experience in which group walkers, over time, developed a sense of belonging, responsibility, and loyalty to their walking group. Pollard et al.’s [55] review of qualitative studies on walking groups draws this out as a “sense of acceptance and belonging” that emerges from all walking groups that meet regularly (p. 6). 

The final sub-theme ‘acting on empathy’ emphasised how being part of the walking group resulted in a developing sense of respect and empathy for others’ physical abilities and needs as well as individual differences. Participants voiced how they were moved by this awareness to take action to help others in the group when needed. While consistent with constructs in the social wellbeing literature, such as being a valuable support provider, appraising oneself as a social resource, and being able to help others in a positive way [60,76,77,78], this sub-theme appears to be a new contribution to the group walking literature and could warrant future study. 

### 4.5. Conceptual Model for Investigating the Effects of NBIs on Individual Social Wellbeing

Given the inconsistency in results regarding social health constructs between quantitative and qualitative NBI studies and the lack of consensus regarding the definitions of core constructs related to the social environment of NBIs, qualitative research can help clarify the relationships of social dimensions for future research. Using conceptual models to link key social influences with social wellbeing outcomes of NBIs, such as GOHWs [31], may also guide future questionnaire development.

**Figure 2:**
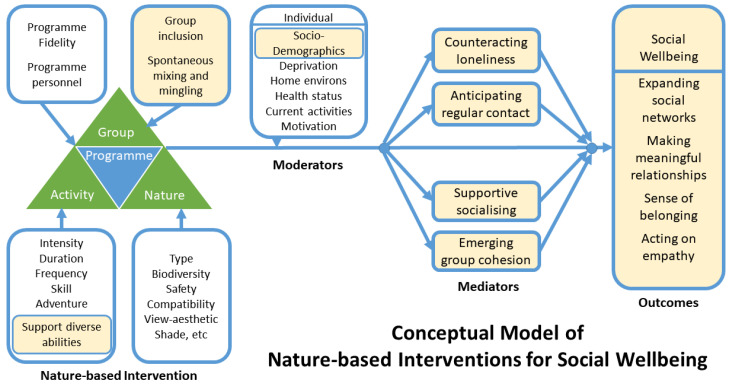
Model for measuring effects of nature-based interventions, such as group outdoor health walks, on individual social wellbeing while accounting for other social dimensions, including social aspects of the programme, socio-demographics, and social experiences as mediators.

Here we use insight from our qualitative thematic analysis and the work of others as discussed above to adapt the overall conceptual model in Figure 1 to create a model which focuses on individual social wellbeing. Figure 2 specifically articulates how the social dimensions of NBIs, such as GOHWs, interact: the components of the programme (i.e., the activity of walking and being part of a group); the mediating factors (e.g., anticipating regular contact); and the individual social wellbeing outcomes (e.g., expanding social networks).

With respect to the components of the programme, our findings suggest conceptualising the ‘group’ aspect of the programme structure as one that facilitates inclusion and spontaneous mixing and mingling amongst walkers. Our findings also show how the structural support for differing abilities enables more people to engage in the activity of walking, which we now incorporate into the ‘activity’ component. 

Associations between variables are subject to modification by individual characteristics. Kwak and Kremers [59] emphasise the important associations that socio-demographics, such as age, gender, and education have with physical activity. To more fully reflect these individual-level moderating factors, we have changed the language to ‘socio-demographics’ within the ‘individual’ box in the conceptual model (Figure 2). 

Four new potential mediators that reflect walkers’ social experiences have been identified and added to the model: mitigating loneliness, anticipating contact, supportive socialising, and group cohesion (Figure 2). The original conceptual model [40] contained only one mediator relating to social aspects, social support (Figure 1), which is not strongly demonstrated in our data. As an example of how the model might influence the selection of questionnaire items in quantitative research, Carron et al.’s [71] scale of group cohesion may be a useful measure along with additional items on loneliness, social isolation, and anticipation of social contact. New items assessing how the group communication and camaraderie supports more physical activity could be derived.

The findings from this study have also helped clarify and identify the components of individual social wellbeing as an outcome for NBIs. In the original conceptual model [40] social wellbeing was one of several biopsychosocial–spiritual wellbeing outcomes of NBIs (Figure 1). In the adapted conceptual model (Figure 2), the outcome of individual social wellbeing could be measured by assessing the extent of social networks, the meaningfulness of relationships, the sense of belonging and the extent to which someone acts on their empathy for others. Select items from existing social wellbeing scales could be combined into a new scale reflecting the specific areas of social wellbeing that are associated with GOHWs [76,78,80]. Questionnaire items to assess one’s sense of belonging, an underassessed aspect of individual social wellbeing [78], may need to be drawn from measures for other social constructs such as social capital, social cohesion, or group cohesion.

### 4.6. Limitations of Research

Our findings were derived from a qualitative case study design incorporating in-depth interviews with a purposive sampling of walkers engaged in a GOHW. While we incorporated techniques to limit social desirability bias (e.g., building rapport, using probe questions following open-ended questions) the material could be influenced by normative pressure. Additionally, while material could be interpreted differently, the data were examined using an iterative process that sought to resolve differences in the thematic mapping of quotes between researchers from different disciplines (environmental psychology, medicine, and geography). 

The majority of participants were female and of limited ethnic diversity and age range. Although this demographic reflects other UK studies on GOHWs [39], nevertheless the findings pertain to a particular setting and to the individuals involved in the walking group being studied. This rich experiential insight, a strength of qualitative research, has informed the development of a conceptual model that will need further assessment in more diverse settings. 

### 4.7. Future Research

Our findings highlight four areas within which social dimensions need to be considered in NBI research: the social character of the intervention itself, the socio-demographics of the walkers, various social-related mediators, and individual social wellbeing as an outcome. We offer a conceptual model (Figure 2) that unpacks the complex interacting social dimensions of NBIs. This conceptual model can inform future research by suggesting the selection of existing validated quantitative scales or the need for newly constructed scales to test derived hypotheses. The specification and quantitative testing of this new conceptual model is critical to further research on group-oriented NBIs that aims to improve individual social health and wellbeing, such as GOHWs, and may also help elucidate the benefits of other social leisure experiences [95]. 

Future research could also fruitfully explore several of the constructs identified. For example, through additional qualitative investigation, the ‘supportive socialising’ and ‘acting on empathy’ dimensions could be further elucidated. Importantly, the model would benefit from further testing within other demographics and contexts, which would facilitate confidence in the generalisability of the model for wider application. 

### 4.8. Implications

Quality greenspaces can encourage people to be outdoors, increasing the likelihood of social interaction and strengthening neighbourhood social ties with others [27,97,98]. As such, there has been emphasis on encouraging older people to use local outdoor spaces and on improving the local area where people live. Improving availability of natural environment spaces alone, however, may not significantly impact social isolation, as in our case study where participants were living within a national park. Older people may be without friends, family, or others to accompany them to make use of the outdoors and there are both real and perceived risks to doing so alone. Thus, interventions, such as GOHWs, that make use of the outdoors for nature-based group activities, offer significant opportunities to reduce social isolation [99]. 

Our findings suggest that these walking groups can promote social interaction and enhance individual social wellbeing as well as facilitate older people to participate in nature. It is, however, important to recognise that they can be detrimental to some people if they are not enjoyed, cannot keep up with others, or have specific and unmet needs [100]. Thus, the structural support for differing abilities, as highlighted in our findings, is an important part of planning any programme of group activity in nature. 

Recent experience in Scotland, UK in delivering NBIs through a network of Green Health Partnerships, including a green prescription programme, points to the value of robust prescription pathways in which adequately resourced third sector link workers are able to access comprehensive and up-to-date information about NBIs available in the community and their suitability for different types of service users [101]. This type of social prescribing is a “way of linking patients in primary care with sources of support within the community” [102] (p. 1). It is increasingly being adopted in the UK as a way to reduce pressure on the National Health Service through supporting service delivery by third-sector community-based organisations [102,103] and include ‘green prescription’ referrals to NBIs such as GOHW programmes. Our study adds support to systematic reviews of the social prescribing literature that show that such programmes have the potential to improve a range of wellbeing outcomes [102,103]. Our conceptual model supports further robust evaluation that is required to demonstrate the cost-effectiveness of social prescribing programmes [102,103]. 

Finally, while we appreciate that the study did not occur during the COVID-19 pandemic, it grapples with social isolation and individual social wellbeing, issues of relevance in the current day environment as we emerge from and take stock of the effects of lockdown(s). Thus, in our assessment, the findings provide insight into how GOHWs as an intervention could help address social isolation and loneliness that have been exacerbated under COVID-19 restrictions.

## 5. Conclusions

The COVID-19 global pandemic and the practices of physical distancing increased social isolation, particularly among our elders. Our results demonstrate how NBIs such as GOHWs can help address isolation and loneliness in older adults in the UK by providing a low-risk social activity that develops and strengthens relationships among members of the walking group. The envelope of the group and the multiple opportunities for spontaneous interaction can counter feelings of loneliness, engender pleasurable anticipation of regular contact with others, and foster group cohesion while also supporting increased physical activity. These in turn can contribute to individual social wellbeing, evidenced by expanded social networks, meaningful relationships, a sense of belonging, and acting on empathy for others. Findings were used to propose a conceptual model of how these social constructs are operating and may inform selection and development of quantitative measures for use in future studies of complex NBIs, such as GOHWs, that can promote individual social wellbeing. 

## Figures and Tables

**Table 1:** Social-related themes associated with a group outdoor health walk.

Themes/Sub-Themes	Description
Theme 1. Programmatic Elements Fostering Engagement	The programme provides the opportunity to be part of a group and a structure that attends to the needs of inexperienced or physically challenged individuals.
Theme 2. Spontaneous Mixing and Mingling	Being part of the group walk fosters casual interpersonal interactions through spontaneous mixing during and after the walk.
Theme 3. Evolving Social Experiences	The spontaneous socialising provides for largely positive social experiences illustrated in four sub-themes.
Counteracting Loneliness	The group walks help combat loneliness and social isolation.
Anticipating Regular Contact	The group walks provide an opportunity for regular contact with other people, breaking up routine, and something to look forward to carrying out.
Supportive Socialising	The group process itself (i.e., the chatting and the camaraderie) helps people join and complete walks they would not have attempted on their own.
Emerging Group Cohesion	Unity and group cohesion emerge in the context of making decisions about the walking.
Theme 4. Achieving Individual Social Wellbeing	Participants demonstrated increased individual social wellbeing illustrated in four sub-themes.
Expanding Social Networks	Participants had a sense of expanding social networks and learning about people in relation to the places around them.
Making Meaningful Relationships	Walkers furthered their ability to make and maintain meaningful relationships and develop friendships.
Sense of Belonging	The sense of belonging to the walking group, including responsibility and loyalty, developed over time.
Acting on Empathy	Being part of the walking group fostered a sense of respect and empathy for others’ physical abilities, needs, and individual differences.

## Data Availability

The (pseudonymized) data presented in this study are available on request from the corresponding author. The data re not publicly available to preserve the confidentiality of participants.
